# *N*-(4-*tert*-Butyl­benz­yl)phthalimide

**DOI:** 10.1107/S1600536809025343

**Published:** 2009-07-04

**Authors:** Jiang-Sheng Li, Jim Simpson, Xun Li

**Affiliations:** a School of Chemistry and Biological Engineering, Changsha University of Science & Technology, Changsha 410004, People’s Republic of China; b Department of Chemistry, University of Otago, PO Box 56, Dunedin, New Zealand

## Abstract

The mol­ecule of the title compound [systematic name: 2-(4-*tert*-butyl­benz­yl)isoindoline-1,3-dione], C_19_H_19_NO_2_, is V-shaped with a dihedral angle of 74.15 (7)° between the mean planes of the phthalimide unit and the benzene ring. The methyl groups of the *tert*-butyl substituent are disordered over two sets of positions, with an occupancy ratio of 0.700 (4):0.300 (4). In the crystal, inter­molecular C—H⋯O hydrogen bonds link adjacent mol­ecules into centrosymmetric dimers. An additional weak C—H⋯O contact, together with weak C—H⋯π and π–π inter­actions [centroid–centroid distance = 3.961 (2) Å] generate a three-dimensional network.

## Related literature

For the synthesis, see: Xin *et al.* (2006 ▶). For related structures, see: Chen *et al.* (2006 ▶); Lü *et al.* (2006 ▶); Warzecha *et al.* (2006*a* ▶,*b* ▶,*c* ▶); Xin *et al.* (2006 ▶).For bond-length data, see: Allen *et al.* (1987 ▶).
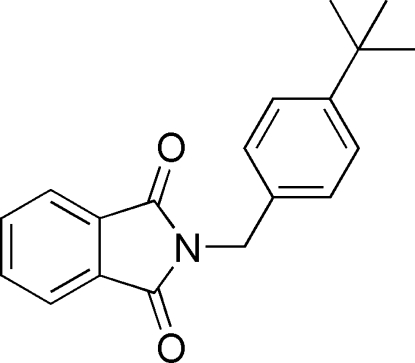


## Experimental

### 

#### Crystal data

C_19_H_19_NO_2_
*M* *_r_* = 293.35
Trigonal, 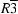

*a* = 37.576 (7) Å
*c* = 6.2970 (16) Å
*V* = 7700 (3) Å^3^
*Z* = 18
Mo *K*α radiation
μ = 0.07 mm^−1^
*T* = 294 K
0.24 × 0.22 × 0.18 mm

#### Data collection

Bruker SMART 1K CCD area-detector diffractometer
Absorption correction: multi-scan (*SADABS*; Sheldrick, 1996 ▶) *T*_min_ = 0.983, *T*_max_ = 0.987
13205 measured reflections
3022 independent reflections
1574 reflections with *I* > 2σ(*I*)
*R*_int_ = 0.060

#### Refinement

*R*[*F* ^2^ > 2σ(*F* ^2^)] = 0.051
*wR*(*F* ^2^) = 0.139
*S* = 1.01
3022 reflections
232 parameters
117 restraints
H-atom parameters constrained
Δρ_max_ = 0.20 e Å^−3^
Δρ_min_ = −0.17 e Å^−3^

### 

Data collection: *SMART* (Bruker, 1997 ▶); cell refinement: *SAINT* (Bruker, 1997 ▶); data reduction: *SAINT*; program(s) used to solve structure: *SHELXS97* (Sheldrick, 2008 ▶); program(s) used to refine structure: *SHELXL97* (Sheldrick, 2008 ▶); molecular graphics: *SHELXTL* (Sheldrick, 2008 ▶) and *Mercury* (Macrae *et al.*, 2006 ▶); software used to prepare material for publication: *SHELXL97*, *enCIFer* (Allen *et al.*, 2004 ▶), *PLATON* (Spek, 2009 ▶) and *publCIF* (Westrip, 2009 ▶).

## Supplementary Material

Crystal structure: contains datablocks gloabl, I. DOI: 10.1107/S1600536809025343/hb5020sup1.cif

Structure factors: contains datablocks I. DOI: 10.1107/S1600536809025343/hb5020Isup2.hkl

Additional supplementary materials:  crystallographic information; 3D view; checkCIF report

## Figures and Tables

**Table 1:** Hydrogen-bond geometry (Å, °)

*D*—H⋯*A*	*D*—H	H⋯*A*	*D*⋯*A*	*D*—H⋯*A*
C6—H6*A*⋯O2^i^	0.93	2.41	3.297 (3)	160
C9—H9*B*⋯O1^ii^	0.97	2.71	3.135 (3)	107
C5—H5*A*⋯*Cg*3^iii^	0.93	2.94	3.771 (4)	149

Symmetry codes: (i) 
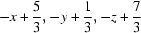
; (ii) 
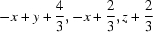
; (iii) 
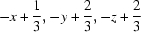
. *Cg*3 is the centroid of the C10–C15 benzene ring.

**Fig. 1.**
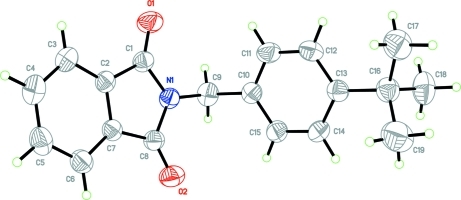
The molecular structure of (I) showing displacement ellipsoids drawn at the 30% probability level and H atoms shown as small spheres of arbitrary radius. Only the major disorder component of the disordered methyl groups is shown.

**Fig. 2.**
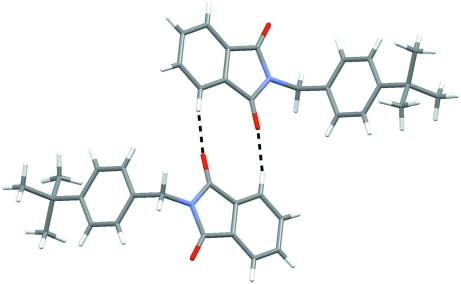
Centrosymmetric dimers of (I) formed by C—H···O hydrogen bonds drawn as dashed lines.

**Fig. 3.**
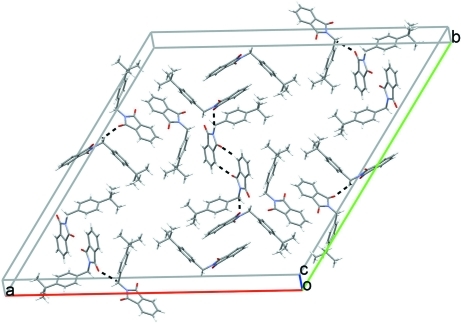
Crystal packing of (I) viewed down the c axis. Hydrogen bonds are drawn as dashed lines.

## supplementary crystallographic information

### Comment

The molecular structure of (I) (Fig. 1) shows that the phthalimide ring system is almost planar, with the dihedral angle between the C2···C7 and N1/C1/C2/C7/C8 rings 1.26 (15) °. The molecule adopts a V-shape with a dihedral angle between the mean planes of the phthalimide group and the benzene ring of 74.12 (7) Å. Bond distances within the molecule are normal (Allen *et al.*, 1987) and similar to those observed in comparable structures (Chen *et al.*, 2006; Lü *et al.*, 2006; Warzecha *et al.*, 2006*a*,b,c; Xin *et al.*, 2006).

In the crystal structure, complementary intermolecular C6—H6a···O2 hydrogen bonds link molecules into dimers (Table 1, Fig. 2). Additional weak C8—H9B···O1 and C—H···π contacts together with π-π interactions between the six-membered phthalimide rings (centroid-centroid separation 3.961 (2) Å; 1/3 - *x*,2/3 - *y*,2/3 - *z*) generate an extensive three-dimensional network structure, Fig. 3.

### Experimental

The title compound was obtained by a literature method (Xin, *et al.*, 2006). Colourless blocks of (I) were grown from an ethanol solution.

### Refinement

The H atoms were positioned geometrically (C—H = 0.93–0.97Å) and refined as riding with *U*_iso_(H) = 1.2 *U*_eq_(C) or 1.5*U*_eq_(methyl C). The three methyl groups of the *tert*-butyl group are disordered over two positions with an occupancy ratio of 0.700 (4):0.300 (4). Restraints were applied to the atomic displacement parameters and interatomic distances for these atoms. *PLATON* (Spek, 2009) reports a solvent accessible voids of total area 164.0 Å^3^ in the structure. However, the low residual electron density does not suggest additional solvent in the structure. This was confirmed using the SQUEEZE procedure (Spek, 2009).

### Crystal data

**Table:**

C_19_H_19_NO_2_	*D*_x_ = 1.139 Mg m^−^^3^
*M**_r_* = 293.35	Mo *K*α radiation, λ = 0.71073 Å
Trigonal, *R*3	Cell parameters from 2125 reflections
Hall symbol: -R 3	θ = 2.9–20.3°
*a* = 37.576 (7) Å	µ = 0.07 mm^−^^1^
*c* = 6.2970 (16) Å	*T* = 294 K
*V* = 7700 (3) Å^3^	Block, colourless
*Z* = 18	0.24 × 0.22 × 0.18 mm
*F*(000) = 2808	

### Data collection

**Table:**

Bruker SMART 1K CCD area-detector diffractometer	3022 independent reflections
Radiation source: fine-focus sealed tube	1574 reflections with *I* > 2σ(*I*)
graphite	*R*_int_ = 0.060
φ and ω scans	θ_max_ = 25.0°, θ_min_ = 1.9°
Absorption correction: multi-scan (*SADABS*; Sheldrick, 1996)	*h* = −44→44
*T*_min_ = 0.983, *T*_max_ = 0.987	*k* = −44→40
13205 measured reflections	*l* = −7→5

### Refinement

**Table:**

Refinement on *F*^2^	Secondary atom site location: difference Fourier map
Least-squares matrix: full	Hydrogen site location: inferred from neighbouring sites
*R*[*F*^2^ > 2σ(*F*^2^)] = 0.051	H-atom parameters constrained
*wR*(*F*^2^) = 0.139	*w* = 1/[σ^2^(*F*_o_^2^) + (0.067*P*)^2^] where *P* = (*F*_o_^2^ + 2*F*_c_^2^)/3
*S* = 1.01	(Δ/σ)_max_ = 0.007
3022 reflections	Δρ_max_ = 0.20 e Å^−^^3^
232 parameters	Δρ_min_ = −0.17 e Å^−^^3^
117 restraints	Extinction correction: *SHELXL97* (Sheldrick, 2008), Fc^*^=kFc[1+0.001xFc^2^λ^3^/sin(2θ)]^-1/4^
Primary atom site location: structure-invariant direct methods	Extinction coefficient: 0.0015 (3)

### Special details

**Table:**

Geometry. All e.s.d.'s (except the e.s.d. in the dihedral angle between two l.s. planes) are estimated using the full covariance matrix. The cell e.s.d.'s are taken into account individually in the estimation of e.s.d.'s in distances, angles and torsion angles; correlations between e.s.d.'s in cell parameters are only used when they are defined by crystal symmetry. An approximate (isotropic) treatment of cell e.s.d.'s is used for estimating e.s.d.'s involving l.s. planes.
Refinement. Refinement of *F*^2^ against ALL reflections. The weighted *R*-factor *wR* and goodness of fit *S* are based on *F*^2^, conventional *R*-factors *R* are based on *F*, with *F* set to zero for negative *F*^2^. The threshold expression of *F*^2^ > σ(*F*^2^) is used only for calculating *R*-factors(gt) *etc*. and is not relevant to the choice of reflections for refinement. *R*-factors based on *F*^2^ are statistically about twice as large as those based on *F*, and *R*- factors based on ALL data will be even larger.

### Fractional atomic coordinates and isotropic or equivalent isotropic displacement parameters (Å^2^)

**Table:**

	*x*	*y*	*z*	*U*_iso_*/*U*_eq_	Occ. (<1)
N1	0.74578 (6)	0.07689 (6)	0.8339 (3)	0.0624 (6)	
O1	0.71269 (6)	0.06224 (6)	0.5108 (3)	0.0914 (7)	
O2	0.78710 (6)	0.11049 (6)	1.1186 (3)	0.0878 (6)	
C1	0.73592 (7)	0.08689 (8)	0.6388 (4)	0.0636 (7)	
C2	0.75936 (7)	0.13258 (7)	0.6282 (4)	0.0592 (6)	
C3	0.76152 (8)	0.15895 (9)	0.4692 (4)	0.0738 (8)	
H3A	0.7463	0.1493	0.3448	0.089*	
C4	0.78736 (9)	0.20036 (10)	0.5043 (5)	0.0857 (9)	
H4A	0.7897	0.2190	0.4003	0.103*	
C5	0.80973 (9)	0.21491 (9)	0.6882 (5)	0.0840 (9)	
H5A	0.8267	0.2431	0.7061	0.101*	
C6	0.80741 (8)	0.18844 (9)	0.8472 (4)	0.0742 (8)	
H6A	0.8225	0.1981	0.9720	0.089*	
C7	0.78172 (7)	0.14702 (8)	0.8123 (4)	0.0586 (6)	
C8	0.77353 (8)	0.11164 (8)	0.9466 (4)	0.0637 (7)	
C9	0.73037 (8)	0.03495 (8)	0.9110 (4)	0.0761 (8)	
H9A	0.7041	0.0167	0.8446	0.091*	
H9B	0.7261	0.0341	1.0632	0.091*	
C10	0.76013 (7)	0.02033 (7)	0.8621 (4)	0.0633 (7)	
C11	0.76087 (8)	0.00447 (8)	0.6666 (5)	0.0779 (8)	
H11A	0.7418	0.0017	0.5640	0.093*	
C12	0.78922 (8)	−0.00735 (8)	0.6194 (4)	0.0769 (8)	
H12A	0.7887	−0.0181	0.4857	0.092*	
C13	0.81833 (8)	−0.00375 (7)	0.7641 (4)	0.0648 (7)	
C14	0.81715 (9)	0.01207 (8)	0.9605 (4)	0.0775 (8)	
H14A	0.8363	0.0151	1.0630	0.093*	
C15	0.78852 (9)	0.02358 (8)	1.0095 (4)	0.0755 (8)	
H15A	0.7885	0.0337	1.1443	0.091*	
C16	0.84998 (8)	−0.01645 (8)	0.7120 (4)	0.0776 (8)	
C17	0.85716 (17)	−0.01769 (19)	0.4734 (6)	0.1105 (16)	0.700 (4)
H17A	0.8317	−0.0366	0.4056	0.166*	0.700 (4)
H17B	0.8769	−0.0265	0.4508	0.166*	0.700 (4)
H17C	0.8674	0.0092	0.4141	0.166*	0.700 (4)
C18	0.83567 (17)	−0.05907 (15)	0.8010 (9)	0.1127 (16)	0.700 (4)
H18A	0.8094	−0.0782	0.7410	0.169*	0.700 (4)
H18B	0.8332	−0.0586	0.9526	0.169*	0.700 (4)
H18C	0.8553	−0.0674	0.7657	0.169*	0.700 (4)
C19	0.89213 (15)	0.01404 (19)	0.8087 (9)	0.1309 (19)	0.700 (4)
H19A	0.8908	0.0112	0.9605	0.196*	0.700 (4)
H19B	0.8992	0.0416	0.7715	0.196*	0.700 (4)
H19C	0.9126	0.0083	0.7544	0.196*	0.700 (4)
C17'	0.8277 (4)	−0.0580 (3)	0.595 (2)	0.125 (3)	0.300 (4)
H17D	0.8080	−0.0786	0.6891	0.188*	0.300 (4)
H17E	0.8474	−0.0657	0.5511	0.188*	0.300 (4)
H17F	0.8138	−0.0556	0.4731	0.188*	0.300 (4)
C18'	0.8704 (4)	−0.0220 (4)	0.9090 (15)	0.106 (3)	0.300 (4)
H18D	0.8506	−0.0456	0.9871	0.160*	0.300 (4)
H18E	0.8806	0.0020	0.9970	0.160*	0.300 (4)
H18F	0.8927	−0.0260	0.8667	0.160*	0.300 (4)
C19'	0.8826 (3)	0.0180 (3)	0.578 (2)	0.115 (3)	0.300 (4)
H19D	0.8698	0.0235	0.4604	0.172*	0.300 (4)
H19E	0.9015	0.0100	0.5249	0.172*	0.300 (4)
H19F	0.8972	0.0423	0.6628	0.172*	0.300 (4)

### Atomic displacement parameters (Å^2^)

**Table:**

	*U*^11^	*U*^22^	*U*^33^	*U*^12^	*U*^13^	*U*^23^
N1	0.0547 (13)	0.0568 (13)	0.0710 (14)	0.0243 (11)	−0.0037 (10)	0.0023 (11)
O1	0.0771 (13)	0.0802 (13)	0.0980 (15)	0.0252 (11)	−0.0323 (11)	−0.0139 (11)
O2	0.0980 (15)	0.0980 (14)	0.0664 (13)	0.0482 (12)	−0.0134 (11)	−0.0046 (10)
C1	0.0501 (15)	0.0666 (18)	0.0722 (18)	0.0279 (14)	−0.0057 (13)	−0.0020 (14)
C2	0.0509 (15)	0.0649 (17)	0.0670 (17)	0.0330 (13)	0.0004 (13)	−0.0011 (14)
C3	0.0691 (18)	0.082 (2)	0.0766 (19)	0.0431 (17)	−0.0010 (14)	0.0084 (16)
C4	0.078 (2)	0.080 (2)	0.105 (2)	0.0436 (18)	0.0076 (18)	0.0199 (17)
C5	0.072 (2)	0.0623 (18)	0.116 (3)	0.0321 (16)	0.0029 (18)	0.0002 (19)
C6	0.0673 (18)	0.0686 (19)	0.088 (2)	0.0349 (15)	−0.0063 (14)	−0.0103 (16)
C7	0.0508 (15)	0.0620 (17)	0.0668 (17)	0.0310 (13)	0.0014 (12)	−0.0037 (13)
C8	0.0624 (16)	0.0716 (18)	0.0616 (17)	0.0369 (15)	−0.0027 (13)	−0.0051 (15)
C9	0.0628 (17)	0.0650 (17)	0.092 (2)	0.0255 (14)	0.0088 (14)	0.0128 (14)
C10	0.0587 (16)	0.0505 (15)	0.0716 (19)	0.0204 (13)	0.0006 (13)	0.0080 (12)
C11	0.0663 (18)	0.0742 (19)	0.082 (2)	0.0265 (15)	−0.0184 (14)	−0.0111 (15)
C12	0.077 (2)	0.0720 (18)	0.0724 (19)	0.0298 (16)	−0.0104 (15)	−0.0166 (14)
C13	0.0677 (17)	0.0519 (15)	0.0676 (17)	0.0244 (13)	0.0006 (14)	0.0029 (12)
C14	0.095 (2)	0.087 (2)	0.0638 (18)	0.0552 (18)	−0.0137 (14)	0.0018 (14)
C15	0.099 (2)	0.0821 (19)	0.0583 (17)	0.0551 (18)	−0.0020 (15)	0.0044 (13)
C16	0.0807 (18)	0.0794 (17)	0.0765 (17)	0.0429 (15)	0.0052 (13)	0.0025 (14)
C17	0.120 (3)	0.137 (3)	0.092 (3)	0.078 (3)	0.021 (2)	0.005 (2)
C18	0.131 (3)	0.106 (3)	0.130 (3)	0.081 (3)	0.026 (3)	0.029 (3)
C19	0.096 (3)	0.148 (4)	0.144 (4)	0.057 (3)	0.003 (3)	−0.036 (3)
C17'	0.124 (5)	0.123 (5)	0.132 (5)	0.065 (4)	0.006 (4)	−0.021 (4)
C18'	0.112 (5)	0.113 (5)	0.113 (5)	0.071 (4)	0.004 (4)	0.014 (4)
C19'	0.098 (4)	0.122 (5)	0.118 (5)	0.052 (4)	0.021 (4)	0.012 (4)

### Geometric parameters (Å, °)

**Table:**

N1—C8	1.391 (3)	C14—C15	1.380 (3)
N1—C1	1.388 (3)	C14—H14A	0.9300
N1—C9	1.464 (3)	C15—H15A	0.9300
O1—C1	1.209 (3)	C16—C19'	1.520 (7)
O2—C8	1.207 (3)	C16—C18	1.519 (4)
C1—C2	1.488 (3)	C16—C18'	1.526 (7)
C2—C7	1.374 (3)	C16—C17	1.531 (4)
C2—C3	1.382 (3)	C16—C17'	1.539 (7)
C3—C4	1.379 (4)	C16—C19	1.542 (5)
C3—H3A	0.9300	C17—H17A	0.9600
C4—C5	1.374 (4)	C17—H17B	0.9600
C4—H4A	0.9300	C17—H17C	0.9600
C5—C6	1.383 (4)	C18—H18A	0.9600
C5—H5A	0.9300	C18—H18B	0.9600
C6—C7	1.378 (3)	C18—H18C	0.9600
C6—H6A	0.9300	C19—H19A	0.9600
C7—C8	1.473 (3)	C19—H19B	0.9600
C9—C10	1.504 (3)	C19—H19C	0.9600
C9—H9A	0.9700	C17'—H17D	0.9600
C9—H9B	0.9700	C17'—H17E	0.9600
C10—C15	1.373 (3)	C17'—H17F	0.9600
C10—C11	1.374 (3)	C18'—H18D	0.9600
C11—C12	1.376 (4)	C18'—H18E	0.9600
C11—H11A	0.9300	C18'—H18F	0.9600
C12—C13	1.377 (3)	C19'—H19D	0.9600
C12—H12A	0.9300	C19'—H19E	0.9600
C13—C14	1.383 (3)	C19'—H19F	0.9600
C13—C16	1.522 (4)		
			
C8—N1—C1	111.9 (2)	C18—C16—C18'	59.5 (5)
C8—N1—C9	123.3 (2)	C19'—C16—C13	106.1 (5)
C1—N1—C9	124.7 (2)	C18—C16—C13	109.3 (3)
O1—C1—N1	124.8 (2)	C18'—C16—C13	113.1 (5)
O1—C1—C2	129.7 (2)	C19'—C16—C17	53.2 (5)
N1—C1—C2	105.5 (2)	C18—C16—C17	107.8 (3)
C7—C2—C3	121.5 (2)	C18'—C16—C17	133.2 (5)
C7—C2—C1	108.1 (2)	C13—C16—C17	113.5 (3)
C3—C2—C1	130.4 (2)	C19'—C16—C17'	113.4 (7)
C4—C3—C2	116.7 (3)	C18—C16—C17'	51.7 (5)
C4—C3—H3A	121.7	C18'—C16—C17'	107.7 (7)
C2—C3—H3A	121.7	C13—C16—C17'	107.9 (5)
C5—C4—C3	122.0 (3)	C17—C16—C17'	61.0 (5)
C5—C4—H4A	119.0	C19'—C16—C19	59.7 (5)
C3—C4—H4A	119.0	C18—C16—C19	109.2 (4)
C4—C5—C6	121.2 (3)	C18'—C16—C19	51.8 (5)
C4—C5—H5A	119.4	C13—C16—C19	110.8 (3)
C6—C5—H5A	119.4	C17—C16—C19	106.2 (3)
C7—C6—C5	116.9 (3)	C17'—C16—C19	141.1 (5)
C7—C6—H6A	121.5	C16—C17—H17A	109.5
C5—C6—H6A	121.5	C16—C17—H17B	109.5
C2—C7—C6	121.7 (2)	H17A—C17—H17B	109.5
C2—C7—C8	108.5 (2)	C16—C17—H17C	109.5
C6—C7—C8	129.8 (2)	H17A—C17—H17C	109.5
O2—C8—N1	123.8 (2)	H17B—C17—H17C	109.5
O2—C8—C7	130.3 (2)	C16—C18—H18A	109.5
N1—C8—C7	106.0 (2)	C16—C18—H18B	109.5
N1—C9—C10	111.05 (19)	H18A—C18—H18B	109.5
N1—C9—H9A	109.4	C16—C18—H18C	109.5
C10—C9—H9A	109.4	H18A—C18—H18C	109.5
N1—C9—H9B	109.4	H18B—C18—H18C	109.5
C10—C9—H9B	109.4	C16—C19—H19A	109.5
H9A—C9—H9B	108.0	C16—C19—H19B	109.5
C15—C10—C11	117.5 (3)	C16—C19—H19C	109.5
C15—C10—C9	121.1 (3)	C16—C17'—H17D	109.5
C11—C10—C9	121.5 (2)	C16—C17'—H17E	109.5
C10—C11—C12	121.3 (2)	H17D—C17'—H17E	109.5
C10—C11—H11A	119.3	C16—C17'—H17F	109.5
C12—C11—H11A	119.3	H17D—C17'—H17F	109.5
C11—C12—C13	122.0 (3)	H17E—C17'—H17F	109.5
C11—C12—H12A	119.0	C16—C18'—H18D	109.5
C13—C12—H12A	119.0	C16—C18'—H18E	109.5
C12—C13—C14	116.0 (3)	H18D—C18'—H18E	109.5
C12—C13—C16	122.2 (2)	C16—C18'—H18F	109.5
C14—C13—C16	121.7 (2)	H18D—C18'—H18F	109.5
C15—C14—C13	122.2 (2)	H18E—C18'—H18F	109.5
C15—C14—H14A	118.9	C16—C19'—H19D	109.5
C13—C14—H14A	118.9	C16—C19'—H19E	109.5
C10—C15—C14	120.9 (2)	H19D—C19'—H19E	109.5
C10—C15—H15A	119.5	C16—C19'—H19F	109.5
C14—C15—H15A	119.5	H19D—C19'—H19F	109.5
C19'—C16—C18	144.5 (5)	H19E—C19'—H19F	109.5
C19'—C16—C18'	108.8 (7)		
			
C8—N1—C1—O1	179.5 (2)	C8—N1—C9—C10	−83.0 (3)
C9—N1—C1—O1	1.8 (4)	C1—N1—C9—C10	94.5 (3)
C8—N1—C1—C2	−0.8 (3)	N1—C9—C10—C15	95.2 (3)
C9—N1—C1—C2	−178.57 (19)	N1—C9—C10—C11	−82.7 (3)
O1—C1—C2—C7	−179.8 (3)	C15—C10—C11—C12	−0.6 (4)
N1—C1—C2—C7	0.5 (2)	C9—C10—C11—C12	177.4 (2)
O1—C1—C2—C3	−1.3 (4)	C10—C11—C12—C13	−0.4 (4)
N1—C1—C2—C3	179.1 (2)	C11—C12—C13—C14	0.6 (4)
C7—C2—C3—C4	0.4 (4)	C11—C12—C13—C16	−179.5 (2)
C1—C2—C3—C4	−178.0 (2)	C12—C13—C14—C15	0.1 (4)
C2—C3—C4—C5	−0.4 (4)	C16—C13—C14—C15	−179.8 (2)
C3—C4—C5—C6	0.2 (4)	C11—C10—C15—C14	1.3 (4)
C4—C5—C6—C7	0.0 (4)	C9—C10—C15—C14	−176.7 (2)
C3—C2—C7—C6	−0.1 (4)	C13—C14—C15—C10	−1.1 (4)
C1—C2—C7—C6	178.5 (2)	C12—C13—C16—C19'	78.0 (6)
C3—C2—C7—C8	−178.8 (2)	C14—C13—C16—C19'	−102.1 (6)
C1—C2—C7—C8	−0.1 (2)	C12—C13—C16—C18	−98.6 (4)
C5—C6—C7—C2	0.0 (4)	C14—C13—C16—C18	81.3 (4)
C5—C6—C7—C8	178.3 (2)	C12—C13—C16—C18'	−162.8 (6)
C1—N1—C8—O2	−178.6 (2)	C14—C13—C16—C18'	17.1 (7)
C9—N1—C8—O2	−0.8 (4)	C12—C13—C16—C17	21.7 (4)
C1—N1—C8—C7	0.8 (3)	C14—C13—C16—C17	−158.4 (3)
C9—N1—C8—C7	178.6 (2)	C12—C13—C16—C17'	−43.8 (7)
C2—C7—C8—O2	179.0 (3)	C14—C13—C16—C17'	136.1 (6)
C6—C7—C8—O2	0.5 (4)	C12—C13—C16—C19	141.1 (4)
C2—C7—C8—N1	−0.4 (2)	C14—C13—C16—C19	−39.0 (4)
C6—C7—C8—N1	−178.9 (2)		

### Hydrogen-bond geometry (Å, °)

**Table:**

*D*—H···*A*	*D*—H	H···*A*	*D*···*A*	*D*—H···*A*
C6—H6A···O2^i^	0.93	2.41	3.297 (3)	160
C9—H9B···O1^ii^	0.97	2.71	3.135 (3)	107
C5—H5A···Cg3^iii^	0.93	2.94	3.771 (4)	149

Symmetry codes: (i) −*x*+5/3, −*y*+1/3, −*z*+7/3; (ii) −*x*+*y*+4/3, −*x*+2/3, *z*+2/3; (iii) −*x*+1/3, −*y*+2/3, −*z*+2/3.
